# Expert Consensus on the Contraindications and Cautions of Foam Rolling—An International Delphi Study

**DOI:** 10.3390/jcm10225360

**Published:** 2021-11-17

**Authors:** Katja Martina Bartsch, Christian Baumgart, Jürgen Freiwald, Jan Wilke, Gunda Slomka, Sascha Turnhöfer, Christoph Egner, Matthias W. Hoppe, Werner Klingler, Robert Schleip

**Affiliations:** 1 Department of Sport Science and Sport (DSS), FAU Erlangen-Nürnberg, 91054 Erlangen, Germany; katja.kb.bartsch@fau.de; 2 Department of Movement and Training Science, University of Wuppertal, 42119 Wuppertal, Germany; baumgart@uni-wuppertal.de (C.B.); freiwald@uni-wuppertal.de (J.F.); 3 Institute of Occupational, Social and Environmental Medicine, Goethe University Frankfurt, 60487 Frankfurt/Main, Germany; wilke@sport.uni-frankfurt.de; 4 Stiftungsuniversität Hildesheim, 31141 Hildesheim, Germany; slomka@uni-hildesheim.de; 5 Diploma Hochschule, 37242 Bad Sooden-Allendorf, Germany; nasakg@aol.com (S.T.); christoph.egner@diploma.de (C.E.); 6 Movement and Training Science, Leipzig University, 04109 Leipzig, Germany; matthias.hoppe@uni-leipzig.de; 7 SRH Hospital, 72488 Sigmaringen, Germany; 8 Experimental Anaesthesiology, Ulm University, 89069 Ulm, Germany; robert.schleip@tum.de; 9 Department of Sports Medicine and Health Promotion, Friedrich Schiller University Jena, 07749 Jena, Germany; 10 Fascia Research Group, Experimental Anesthesiology, Ulm University, 89069 Ulm, Germany; 11 Conservative and Rehabilitative Orthopedics, Department of Sport and Health Sciences, Technical University of Munich, 80809 Munich, Germany

**Keywords:** myofascial, fascia, self-myofascial release, massage

## Abstract

Background: Foam rolling is a type of self-massage using tools such as foam or roller sticks. However, to date, there is no consensus on contraindications and cautions of foam rolling. A methodological approach to narrow that research gap is to obtain reliable opinions of expert groups. The aim of the study was to develop experts’ consensus on contraindications and cautions of foam rolling by means of a Delphi process. Methods: An international three-round Delphi study was conducted. Academic experts, defined as having (co-) authored at least one PubMed-listed paper on foam rolling, were invited to participate. Rounds 1 and 2 involved generation and rating of a list of possible contraindications and cautions of foam rolling. In round 3, participants indicated their agreement on contraindications and cautions for a final set of conditions. Consensus was evaluated using a priori defined criteria. Consensus on contraindications and cautions was considered as reached if more than 70% of participating experts labeled the respective item as contraindication and contraindication or caution, respectively, in round 3. Results: In the final Delphi process round, responses were received from 37 participants. Panel participants were predominantly sports scientists (*n* = 21), physiotherapists (*n* = 6), and medical professionals (*n* = 5). Consensus on contraindications was reached for open wounds (73% agreement) and bone fractures (84%). Consensus on cautions was achieved for local tissue inflammation (97%), deep vein thrombosis (97%), osteomyelitis (94%), and myositis ossificans (92%). The highest impact/severity of an adverse event caused by contraindication/cautions was estimated for bone fractures, deep vein thrombosis, and osteomyelitis. Discussion: The mechanical forces applied through foam rolling can be considered as potential threats leading to adverse events in the context of the identified contraindications and cautions. Further evaluations by medical professionals as well as the collection of clinical data are needed to assess the risks of foam rolling and to generate guidance for different applications and professional backgrounds.

## 1. Background

Foam rolling is a type of self-massage using soft or rigid foam rolls [1,2,3,4]. Despite its broad application and popularity in medicine and sports [5,6], a gap of knowledge exists regarding contraindications and cautions, when aiming to integrate foam rolling as a therapeutic or training tool into practice [7]. In fact, a large body of evidence describes the effects and mechanisms of foam rolling [4,8,9,10,11] but less is known regarding contraindications and cautions [12,13]. Table 1 shows a PubMed literature search that underlines this lack of research.

Guidelines advise clinicians to evaluate the underlying pathology by checking for alarm signals during physical examination and assessment of the patient’s medical history [14,15,16]. Such alarm signals can be labelled as contraindications and cautions. It is recommended that the existence of generally accepted contraindications and cautions (in addition to the related information of health service providers about these) contributes to an improved client assessment by clinicians [17].

As indicated by Table 1, a literature search, revealed only one paper that comments on the clinical standards and contraindications/cautions within the broader context of foam rolling [13]. The authors of that study considered the therapeutic massage literature [18,19,20,21,22,23,24] to produce an initial list of potential contraindications and cautions as empirical evidence is scarce. While said paper is commenting on the contraindications and cautions of roller massage practices, the list of contraindications and cautions can serve as a valuable starting point for more focused investigations.

Besides the collection of clinical data as the gold standard model, obtaining a reliable opinion consensus of experts represents another way of gaining insight into potential contraindications and cautions [25]. In view of the scarcity of papers targeting contraindications and cautions of foam rolling, our study aimed to provide initial data on expert opinions using a Delphi process, which has been widely used across numerous disciplines to seek expert opinions in an iterative structured manner [26,27,28].

## 2. Methods

### 2.1. Ethics and General Approach

The international 3-round Delphi study was conducted between February and May 2021. Ethics approval was obtained from the local ethic committee (Ethics Committee of the Diploma University of Applied Sciences, Bad Sooden-Allendorf, Germany, Ref. no. EB 1003). All participants provided written informed consent. The study was registered with the German Clinical Trials Register (DRKS, number DRKS00025347).

The Delphi method is a standardized technique to interactively and iteratively discuss, form and pool the opinions of several individuals [25,29]. The optimal methodological approach defines several specific criteria for reaching consensus a priori [30,31]. Throughout the process, anonymity between participants as well as controlled feedback constitute key features of the method [32].

### 2.2. Recruitment of Panel Members

**Figure 1:**
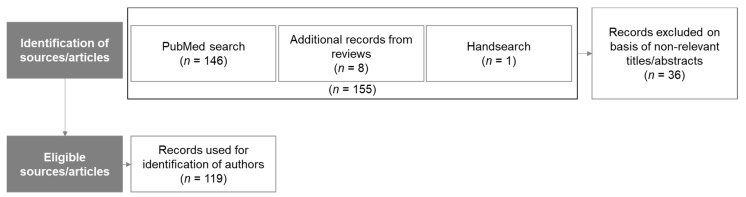
Overview of selection process for relevant publications.

Academic experts were invited to participate in the study. Inclusion criterion for being considered as an academic expert was the publication of at least one peer-reviewed paper on foam rolling as first author or co-author. Previous Delphi studies on contraindications and risks of therapies and interventions achieved consensus with 30 participants [26,33]. Therefore, the authors aimed to have at least 30 respondents for each round. As administered and recommended by prior research [34,35], publicly available bibliographic information from our initial PubMed search (Table 1) was used to identify academic experts. The 146 sources found by the PubMed search were evaluated through a title/abstract screening conducted by three of the authors. Thirty-six records were excluded because of lacking foam rolling content. Furthermore, 8 relevant articles from reviews/meta-analyses and 1 reference through hand search were manually added, leading to a total number 119 eligible sources/articles. The most important criteria for inclusion were an indication of actual experience of the authors/experts with foam rolling in addition to a demonstrated ability to participate in a written discussion in English language about it. Exclusion criteria included lack of any publicly available written material from the expert related to foam rolling, and non-responsiveness in relation to our written invitation. Figure 1 gives an overview of the identification process of relevant publications.

**Figure 2:**
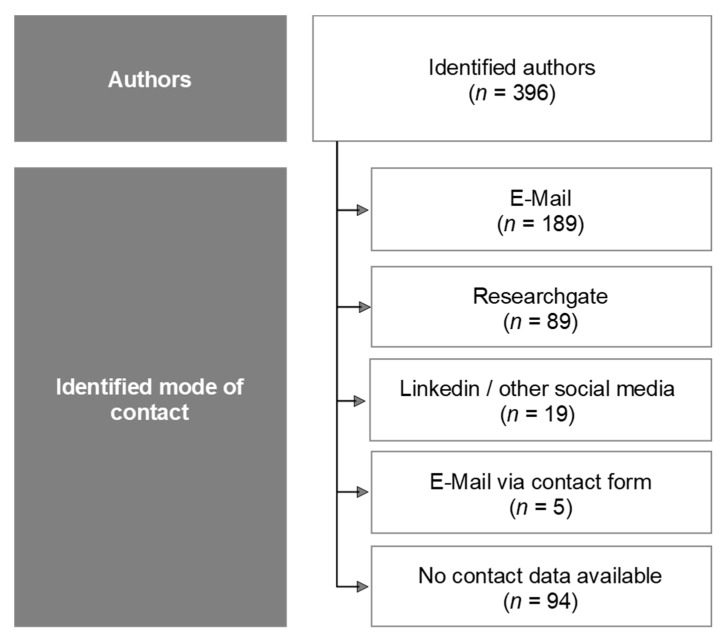
Overview of possible authors/academic experts and contact information.

All authors (including co-authors) of the 119 records were documented, which resulted in a total of 396 names. Then, the Internet was searched to obtain contact data of the said 396 authors. Figure 2 shows an overview of the search results. Experts were contacted and invited to take part in the Delphi process via email and online contact forms and social networks with a description of the study goal and process. No additional screening as for potential conflicts of interest or a representative composition of age, gender, nationalities and professions was conducted.

#### 2.2.1. Delphi Process Rounds and Agreement

**Figure 3:**
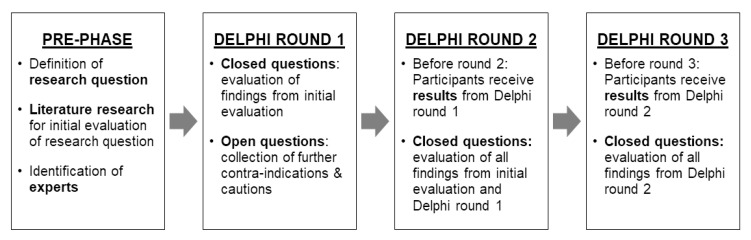
Rounds of the Delphi process.

The present study included a preparatory phase as well as three rounds of the actual Delphi process (see Figure 3).

#### 2.2.2. Preparatory Phase

Survey development and refinement of the research question was conducted by the Study Steering Group. This group consisted of the study researchers, who met at key stages to discuss and make decisions on protocol design, participant recruitment, data analysis, and study conduct. The Study Steering Group compiled and discussed all participant comments after each round. This group consisted of 10 academic researchers of which 5 members had published peer reviewed articles themselves within the field of foam rolling and 4 used foam rolling regularly in their therapeutic work. All group members were primarily located in Germany. Potential conflicts of interest are declared below.

A literature search was performed in order to obtain an initial list of potential contraindications and cautions for foam rolling. Participants were identified and contacted as described above.

#### 2.2.3. Delphi Process Round 1

In round 1, the initial list of contraindications and cautions obtained from the literature was presented to the experts. Participants were asked to rate whether they agreed that the respective line item has to be considered as contraindication/caution on a 6-point Likert scale (1 = strongly disagree, 6 = strongly agree). Participants had the option to give a short explanation of their rating in open text boxes. 

Previous studies have used the first round of a 3-round Delphi process to brainstorm for important factors using open questions [36,37,38]. Therefore, the possibility for experts to brainstorm further possible contraindications and cautions in an open question format was added and no distinction between contraindications and cautions in round 1 was made. In this study, the terms contraindication and caution were defined as follows: relating to foam rolling, a contraindication was defined as a condition or factor that makes foam rolling inadvisable. Contraindications can be absolute (i.e., life threatening) or relative (i.e., higher risk of complications in which benefits may outweigh risks). A caution was defined as a condition that increases the risk for a serious short- or long-term adverse reaction. It can furthermore be defined as a “cautionary or warning symptom that warrants consideration of a need for screening”. For both contraindications and cautions of foam rolling, the context as the person’s age, gender, medical history etc., have to be considered [39].

After a 10-day answer period, results were evaluated by the Study Steering Group and provided to the participants. Participants were asked to read the results from round 1 before the next survey round.

#### 2.2.4. Delphi Process Round 2

In round 2, the synthesized contraindications and cautions were evaluated in a closed question format by the participating experts (Table 2). 

Previous Delphi studies have used the second round of a 3-round Delphi process to narrow down the original list from round 1 by using pre-defined criteria for dropping items, thereby reducing the list to a manageable size [32,36,37,38]. The same approach was applied by asking participants to rate whether they agreed that the respective line item had to be considered as contraindication/caution on a 6-point Likert scale (1 = strongly disagree, 6 = strongly agree). Participants had the opportunity to provide a short explanation of their rating but no possibility to add new contraindications or cautions. Identical to round 1, participants did not distinguish between contraindications and cautions—both were treated the same.

Defining consensus criteria a priori is considered a quality indicator for Delphi studies. A systematic review on the definition of consensus found that the most common definition for consensus was percent agreement, with the median threshold for the determination of consensus being 75% (range: 50–97%) [32]. Therefore, it was predefined that only contraindications/cautions that at least 75% of participating experts agreed with (Likert score 5 or more) were further considered for round 3 of the Delphi process.

Statistical and qualitative results of round 2 were provided to all participants before the start of round 3. As in the previous round, participants were asked to read the results before answering the survey of round 3.

#### 2.2.5. Delphi Process Round 3

In round 3, the contraindications/cautions deemed relevant in round 2 were reviewed (Table 2). Participants had to label each of the presented items as either “contraindication” or “caution” or “neither contraindication nor caution” in a closed question format.

Consensus on contraindications was defined a priori if more than 70% of participating experts labeled the respective item as contraindication. Consensus on cautions was predefined if more than 70% of participating experts labeled the line item as contraindication or caution. All line items that did not meet these criteria were predefined as neither contraindication nor caution.

Additionally, participants were asked to indicate the estimated impact/severity of an adverse event caused by the respective contraindication/caution. In medicine and health sciences, quality of life has been used as a measure to identify the range of problems that can affect patients and to help them anticipate and understand the consequences of a condition and its treatment [40]. Therefore, the estimated impact/severity of potential adverse events by the degree to which the quality of life would be influenced by someone experiencing the respective contraindication/caution on a 7-point Likert scale was measured (1 equaling very bad quality of life, 7 equaling excellent quality of life).

Furthermore, participants were given the option to argue against the drop of items after round 2. In an open format text field, experts could outline as to why they would consider such items as contraindication or caution. 

The results of round 3 were predefined as end point of the Delphi process.

**Figure 4:**
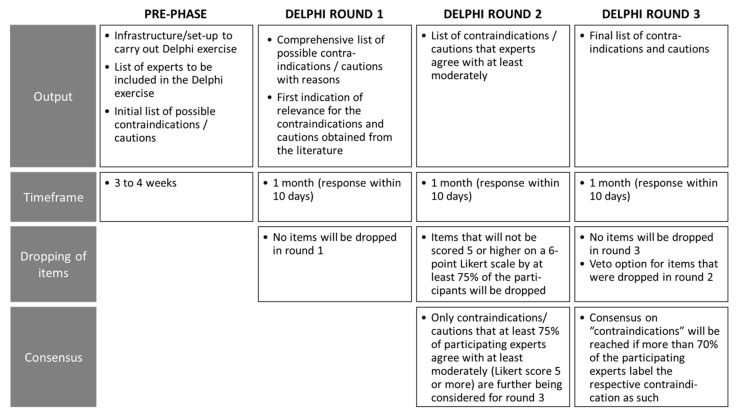
Outputs, planned timeframes, items to be dropped and requirements for consensus of each Delphi process round.

The outputs, planned timeframes, items to be dropped and requirements for consensus of each round are depicted in Figure 4.

#### 2.2.6. Data Processing

Study data were collected using an electronic questionnaire (SurveyMonkey Europe UC, Dublin, Ireland) between March and May 2021. After seven and ten workdays, reminders were sent to participants in each round. Data collection was carried out anonymously [32].

Descriptive statistical analyses were performed to evaluate the closed-question Likert scale. All quantitative statistical analyses were made with Excel (Microsoft Corporation, Redmond, WA, USA). Open text responses were compiled by the Study Steering Group.

## 3. Results

**Figure 5:**
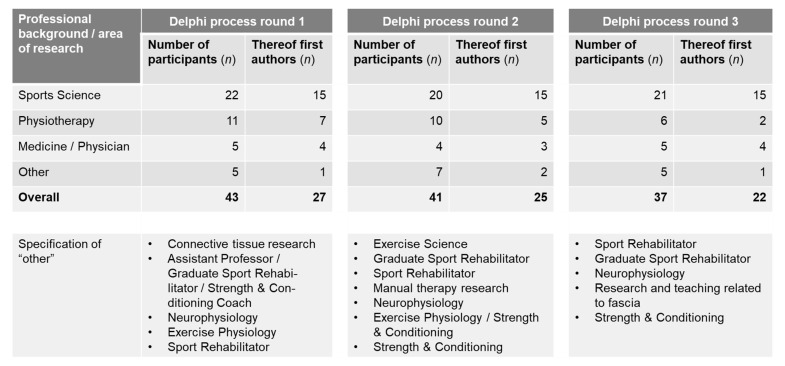
Overview of participant characteristics.

Forty-eight experts volunteered to participate. Responses were received from 43 participants in Delphi process round 1 (10.9% response rate of all identified experts; 89.6% response rate of experts who provided consent), from 41 participants in Delphi process round 2 (10.4%/85.4% response rate), and from 37 participants (9.3%/77.1% response rate) in Delphi process round 3, respectively. Experts were mainly sports scientists (*n* = 21 in round 3) followed by physiotherapists (*n* = 6) and medical professionals (*n* = 5). The nationalities represented among these included USA (9), Germany (8), United Kingdom (7), Spain (6), France (2), as well as 1 person each from Australia, Austria, Brazil, Canada, Greece, Ireland, Italy, Japan, Poland, Taiwan, and Turkey. An overview of the characteristics of study participants for each round is depicted in Figure 5.

After Delphi process round 1, the Study Steering Group agreed that participant comments supported to subdivide one existing statement and add 6 conditions (Table 2). A total of 38 conditions were submitted to round 2. After round 2, consensus was achieved for three conditions: local tissue inflammation/open wounds (85.0% percentage of experts rating agree/strongly agree), bone fracture or myositis ossificans (82.5%), deep vein thrombosis or osteomyelitis (82.5%). After round 2, the Study Steering Group discussed and agreed to subdivide the three conditions, thus, a total of 6 conditions were taken into round 3 (Table 2).

In round 3, consensus on contraindications of foam rolling was reached for open wounds (73.0% percentage agreement; percentage of experts rating the condition as contraindication) and bone fractures (83.8%). Consensus on cautions was achieved for local tissue inflammation, myositis ossificans, deep vein thrombosis, and osteomyelitis, with very high percentage agreement (percentage of experts rating the condition as contraindication or caution) ranging from 91.9% to 97.3%. Table 3 provides an overview of participants’ answers in round 3.

**Figure 6:**
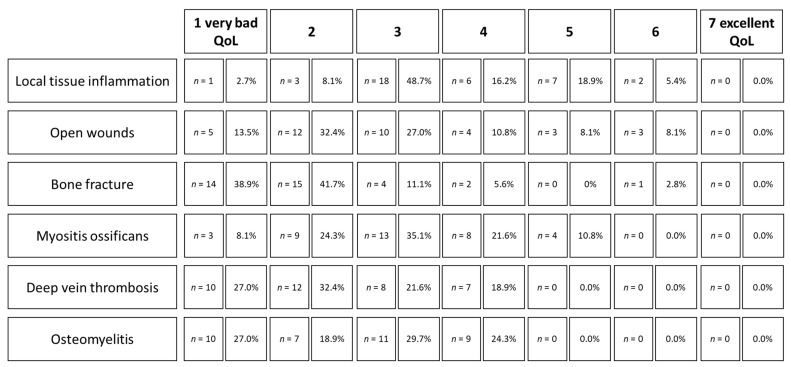
Estimated impact/severity of an adverse event (defined as quality of living; QoL) caused by contraindications/cautions.

Participants indicated the highest estimated impact/severity of an adverse event caused by contraindication/cautions for bone fractures, deep vein thrombosis, and osteomyelitis represented by the highest ratings for very bad quality of living (QoL) for these conditions. Figure 6 depicts the detailed answers of participants on estimated impact/severity in round 3.

**Figure 7:**
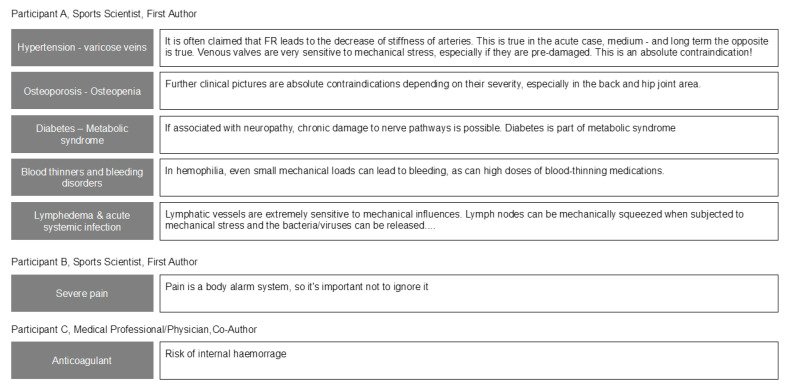
Arguments against dropping of items after Delphi process round 2.

In round 3, three participants made use of the option to argue against the dropping of items after round 2. An overview of their responses is given in Figure 7.

**Figure 8:**
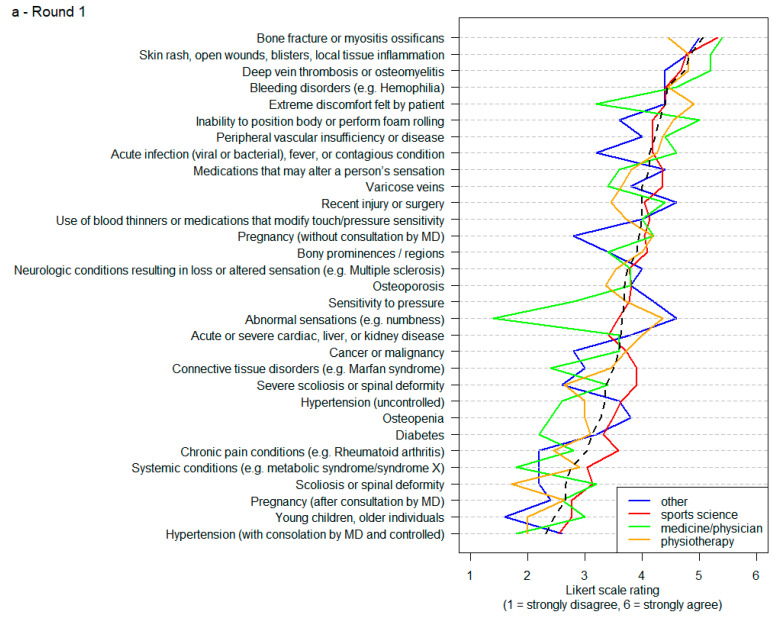

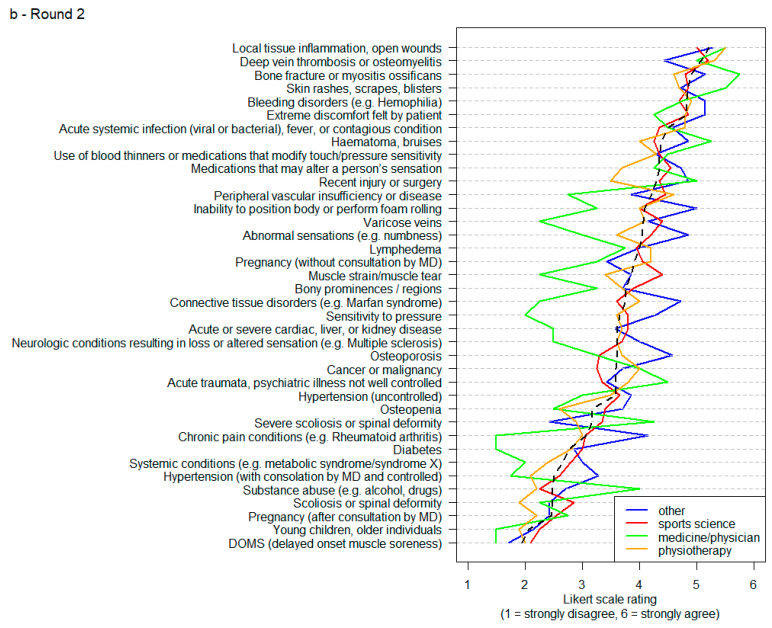
Participants’ responses in Delphi according to professional background in round 1 (**a**) and round 2 (**b**). Note: The black line indicates the mean.

When conducting sub-analyses for the different professional backgrounds (i.e., sports science, physiotherapy, medicine/physicians, other), slight differences in opinion emerged. After Delphi process round 2, several conditions would have moved on to Delphi process round 3 according to the evaluation of medical professionals/physicians (skin rashes, scrapes, blisters, hematoma, bruises), sports scientists (skin rashes, scrapes, blisters), and professionals from other backgrounds (uncontrolled hypertension, bleeding disorders). Furthermore, after Delphi process round 3, consensus on contraindications was assessed slightly differently among the professional groups (medicine/physicians: local tissue inflammation, bone fracture, osteomyelitis; sports scientists: bone fracture; physiotherapists: open wounds, bone fracture, deep vein thrombosis, osteomyelitis; other backgrounds: open wounds, bone fractures, myositis ossificans, deep vein thrombosis). Figure 8a,b provide detailed information about participants’ responses sorted by professional background across Delphi process rounds 1 and 2.

## 4. Discussion

This is the first study to gain international expert consensus on contraindications and cautions of foam rolling. The agreement for the final set of conditions was high (with at least 73% agreement) for contraindications and very high (with at least 92% agreement) for cautions. Our results provide implications for professionals in sports, physical therapy, and medicine: researchers, coaches, and clinicians should consider potential risks of foam rolling in future research and clinical practice.

Our main finding was that bone fractures and open wounds were classified as contraindications of foam rolling by the panel experts, while local tissue inflammation, myositis ossificans, osteomyelitis and deep vein thrombosis were categorized as precautions.

When applying foam rolling to musculoskeletal tissues, mechanical forces are administered. Similarly, the introduction of mechanical forces is used by clinicians and coaches to promote tissue healing, as musculoskeletal tissues respond and adapt to their mechanical environment [41]. As the specific effects of foam rolling on underlying mechanistic (as well as neural) mechanisms are still unclear [8], one can draw information from the neighboring fields of physical and manual therapy in order to evaluate the contraindications and cautions identified by panel experts.

### 4.1. Open Wounds and Local Tissue Inflammation

Open wounds range from abrasions, minor skin incision or tears to wounds with extensive tissue damage or loss. Wound healing includes sequential, yet overlapping phases, including hemostasis, inflammation, proliferation, and remodeling [42,43]. Despite a great body of literature, many underlying pathophysiological processes of wound healing are still unknown [44]. It appears evident that the healing process in the initial phases of hemostasis should not be disturbed through mechanical disruption (as would be the case when foam rolling is applied). A more complicated picture emerges once the initial wound closure is complete and the subsequent inflammatory processes commence. When looking to evaluate the effect of myofascial release therapies on wound healing and inflammatory processes, recent studies suggest that soft tissue inflammation may be reduced by modalities such as soft tissue massage [41]. A range of in vitro studies has looked at fibroblasts’ response to mechanical forces, i.e., myofascial release techniques. Findings of these studies included reduction of wound size, increased collagen density, and improved cell deposition at the wound site by means of fibroblast proliferation [45,46]. Another in vitro study showed that therapeutic soft tissue massage may reduce pro-inflammatory cytokines such as IL-6 and IL-8 [47]. However, the effect of myofascial release techniques on certain inflammatory mediators seems to be dose-dependent: by varying the parameters of the applied biomechanical stimuli, myofascial release can be both an activator and an inhibitor of fibroblast-inflammation [48]. While these in vitro findings are promising evidence in favor of myofascial release techniques in general and consequently for modalities such as foam rolling in particular, they cannot easily be correlated with clinical outcomes. In vivo studies on human wound healing are needed to improve our understanding of the pathogenesis of wound healing as well as the influence and dose-response relationships of myofascial modalities such as foam rolling on its underlying molecular processes.

### 4.2. Bone Fractures

The healing process of a bone fracture progresses from an initial inflammatory phase (0–3 days after injury) to the formation of a soft and then a hard callus (4 days to months after injury), and finally to bony remodeling (months to years after an injury) [42,49]. During initial callus formation, maximal possible rigidity (i.e., stabilization) is desirable. Once the mineralized callus is formed, the application of mechanical strain can influence bone healing positively. According to experimental data, the optimal mechanical strain should range between 100 and 2000 microstrain (during normal walking, bones experience strains in the range of 1000 microstrain). When the applied strain exceeds the allowable strain, hard callus will not form. Clinical guidelines entail recommendations for progressive load bearing [50]. However, as there is no methodology or device to reliably guide or apply the ideal amount of strain, and as further parameters such as type and location of bone, age, and hormonal status are to be considered individually, no absolute parameters of strain magnitudes and frequencies exist [42,50]. Apart from the lack of absolute strain recommendations for all patients, measuring the definite strain applied through foam rolling is not possible with current foam rolling tools. Accordingly, the application of foam rolling may well impair fracture healing by applying too much strain. Panelists’ categorization of bone fractures as contraindications therefore presents as plausible and reasonable. Future studies are needed to provide answers on absolute strain parameters and lead to the development of tools that allow for targeted load application.

### 4.3. Deep Vein Thrombosis

Deep vein thrombosis and pulmonary embolism are collectively referred to as venous thromboembolism and are associated with substantial morbidity and mortality. Strong risk factors include surgery, immobilization, and cancer. Anticoagulants constitute the primary approach to therapy [51,52]. 

The application of mechanical forces through modalities such as manual therapy or massage can potentially mechanically dislodge a thrombus, as reported by various case reports [53,54,55,56]. The most recent of these case studies reported that leg massage during pregnancy with unrecognized deep vein thrombosis can dislodge thrombi, leading to a life-threatening pulmonary embolism. Massage of the lower extremity in cases of deep venous thrombosis is therefore contraindicated according to the authors [56]. Consequently, as foam rolling potentially leads to similar mechanical forces as massage, the panelists’ evaluation of deep vein thrombosis as a contraindication of foam rolling is in line with the above mentioned case reports and studies. 

As many individuals with a first episode of venous thromboembolism will have a recurring event [57], it remains unclear whether patients with a history of deep vein thrombosis should consider modalities like foam rolling with progression of time, as current therapy guidelines do not include respective recommendations [58]. Future studies will need to gauge the risk of treatments such as foam rolling for former patients and populations at risk.

### 4.4. Osteomyelitis

Osteomyelitis is an inflammation of bone tissue caused by an infectious agent. About half of all cases in adult patients are post-traumatic, meaning that the disease occurs at the wound site after surgery, open fractures, or other injuries. In acute post-traumatic as well as in chronic osteomyelitis, surgery with debridement and antimicrobial/antibiotic therapy are recommended. Drainage and wound closure are further important steps of infection control [59,60]. The use of external fixators, sometimes in combination of negative pressure closed drainage is another example of the condition’s clinical management [60]. Given the nature of the disease and its treatment, experts’ evaluation of osteomyelitis as a contraindication of foam rolling presents as apparent and logical.

### 4.5. Myositis Ossificans

Myositis ossificans is a benign, ossifying soft-tissue mass, typically occurring within skeletal muscle [61]. Symptoms include localized pain and swelling as well as loss of range of motion. The exact pathophysiology of the condition is still poorly understood [61]. The guiding principle in treatment of myositis ossificans is to minimize symptoms and to restore function. Any modalities that may increase the risk for bleeding are to be avoided, particularly in the early phases of the condition. Other modalities which have been described to improve some muscular contusions have been described to exacerbate symptoms if performed too soon or aggressively [61,62]. Avoiding pressure through foam rolling in order to avoid further hematoma or contusions is in line with these recommendations. Future research is needed to describe and quantify the specific effects of foam rolling on this condition.

### 4.6. Limitations

Although the Delphi process resulted in high agreement among academic experts, there are a few limitations. While our results represent participants’ perception and awareness about contraindications and cautions of foam rolling, they do not indicate the actual medical risks of foam rolling. Further evaluations by medical professionals as well as the collection of clinical data are needed in the future to assess the risks of foam rolling and to generate guidance for different applications and professional backgrounds. Our results may indicate potential directions and avenues for future research and can assist in selecting the scope for future controlled studies.

Although a diverse range of professional backgrounds among panelists was represented, a large proportion (56.8% in round 3) of participants had a sports science background. This mirrors our approach of recruitment, as the current majority of the foam rolling literature originates from the realm of sports science. 

Further, the varieties in answers between professional groups indicate that the awareness for contraindications and cautions of foam rolling may differ in various settings. This may be caused by diverse experiences and observations by the experts. Within this context it is important to note that a clear definition of foam rolling regarding applied forces, velocity, and duration [63,64,65] is still missing. Furthermore, various kinds of foam rolling tools—different surface types [66], densities [67,68], and incorporation of vibration elements [69,70,71]—are currently being discussed in the literature. Experiences with different characteristics of these parameters may have influenced experts’ ratings and assessments.

## 5. Conclusions

This Delphi study outlines potential contraindications and cautions of foam rolling of which none have been reported within clinical trials yet. Open wounds and bone fractures were classified as contraindications of foam rolling by the panel experts, while local tissue inflammation, deep vein thrombosis, myositis ossificans, and osteomyelitis were categorized as cautions. Future work should develop evidence-based data on contraindications and cautions of foam rolling for different conditions as well as different professional settings.

## Figures and Tables

**Table 1:** PubMed literature search on contraindications and cautions of foam rolling. The search was limited to human subjects and performed on 2 November 2020.

Search Steps	Search Terms	Search Results
1	“foam roll”, “foam rolling”, or “foam roller” combined by Boolean logic (“OR”)	146
2	“contraindication*”, “caution*”, “precaution*”, “red flag*”, or “yellow flag*” combined by Boolean logic (“OR”)	75.613
1 AND 2	combined by Boolean logic (“AND”)	1

**Table 2:** Conditions to be rated throughout Delphi process rounds 1–3.

Item	Round 1	Round 2	Round 3
1	Abnormal sensations (e.g., numbness)	Abnormal sensations (e.g., numbness)	Local tissue inflammation
2	Hypertension (with consolation by MD and controlled)	Hypertension (with consolation by MD and controlled)	Open wounds
3	Hypertension (uncontrolled)	Hypertension (uncontrolled)	Bone fracture
4	Diabetes	Diabetes	Myositis ossificans
5	Systemic conditions (e.g., metabolic syndrome/syndrome X)	Systemic conditions (e.g., metabolic syndrome/syndrome X)	Deep vein thrombosis
6	Chronic pain conditions (e.g., Rheumatoid Arthritis)	Chronic pain conditions (e.g., Rheumatoid Arthritis)	Osteomyelitis
7	Connective tissue disorders (e.g., Marfan syndrome)	Connective tissue disorders (e.g., Marfan syndrome)	
8	Pregnancy (without consultation by MD)	Pregnancy (without consultation by MD)	
9	Pregnancy (after consultation by MD)	Pregnancy (after consultation by MD)	
10	Inability to position body or perform foam rolling	Inability to position body or perform foam rolling	
11	Sensitivity to pressure	Sensitivity to pressure	
12	Extreme discomfort felt by patient	Extreme discomfort felt by patient	
13	Medications that may alter a person’s sensation	Medications that may alter a person’s sensation	
14	Use of blood thinners or medications that modify touch/pressure sensitivity	Use of blood thinners or medications that modify touch/pressure sensitivity	
15	Bleeding disorders (e.g., Hemophilia)	Bleeding disorders (e.g., Hemophilia)	
16	Young children, older individuals	Young children, older individuals	
17	Skin rash, open wounds, blisters, local tissue inflammation	Hematoma, bruises	
18	Acute infection (viral or bacterial), fever, or contagious condition	Skin rashes, scrapes, blisters	
19	Bony prominences/regions	Local tissue inflammation, open wounds	
20	Bone fracture or myositis ossificans	Acute systemic infection (viral or bacterial), fever, or contagious condition	
21	Recent injury or surgery	Lymphedema	
22	Scoliosis or spinal deformity	Muscle strain/muscle tear	
23	Severe scoliosis or spinal deformity	DOMS (delayed onset muscle soreness)	
24	Osteoporosis	Bony prominences/regions	
25	Osteopenia	Bone fracture or myositis ossificans	
26	Neurologic conditions resulting in loss or altered sensation (e.g., Multiple Sclerosis)	Recent injury or surgery	
27	Acute or severe cardiac, liver or kidney disease	Scoliosis or spinal deformity	
28	Peripheral vascular insufficiency or disease	Severe scoliosis or spinal deformity	
29	Deep vein thrombosis or osteomyelitis	Osteoporosis	
30	Varicose Veins	Osteopenia	
31	Cancer or malignancy	Neurologic conditions resulting in loss or altered sensation (e.g., Multiple Sclerosis)	
32		Acute traumata, psychiatric illness not well controlled	
33		Acute or severe cardiac, liver or kidney disease	
34		Peripheral vascular insufficiency or disease	
35		Deep vein thrombosis or osteomyelitis	
36		Varicose Veins	
37		Cancer or malignancy	
38		Substance abuse (e.g., alcohol, drugs)	

**Table 3:** Delphi process round 3 responses. Figures highlighted in grey met the a priori defined criteria for contraindications/cautions.

	Contraindication		Caution		Neither Contraindication nor Caution		Contraindication + Caution			Result According to Valuation Logic
	*n*	%	*n*	%	*n*	%	*n*	%	Sum	
Local tissue inflammation	15	41	21	57	1	3	36	97	37	Caution
Open wounds	27	73	8	22	2	5	35	95	37	Contraindication
Bone fracture	31	84	4	11	2	5	35	95	37	Contraindication
Myositis ossificans	20	54	14	38	3	8	34	92	37	Caution
Deep vein thrombosis	24	65	12	32	1	3	36	97	37	Caution
Osteomyelitis	22	61	12	33	2	6	34	94	36	Caution

## Data Availability

The datasets that will be used and/or analyzed during the current study will be available from the corresponding author on reasonable request.
